# Gold Nanoparticles and Graphene Oxide Flakes Enhance Cancer Cells’ Phagocytosis through Granzyme-Perforin-Dependent Biomechanism

**DOI:** 10.3390/nano11061382

**Published:** 2021-05-24

**Authors:** Mohsen S. Al-Omar, Majid Jabir, Esraa Karsh, Rua Kadhim, Ghassan M. Sulaiman, Zainab J. Taqi, Khawla S. Khashan, Hamdoon A. Mohammed, Riaz A. Khan, Salman A. A. Mohammed

**Affiliations:** 1 Department of Medicinal Chemistry and Pharmacognosy, College of Pharmacy, Qassim University, Qassim 51452, Saudi Arabia; m.omar@qu.edu.sa (M.S.A.-O.); ham.mohammed@qu.edu.sa (H.A.M.); 2 Medicinal Chemistry and Pharmacognosy Department, Faculty of Pharmacy, Jordan University of Science and Technology, Irbid 22110, Jordan; 3 Department of Applied Sciences, University of Technology, Baghdad 10066, Iraq; esraa.h97@yahoo.com (E.K.); ruajali@yahoo.com (R.K.); 100293@uotechnology.edu.iq (Z.J.T.); 100082@uotechnology.edu.iq (K.S.K.); 4 Department of Pharmacognosy, Faculty of Pharmacy, Al-Azhar University, Cairo 11371, Egypt; 5 Department of Pharmacology and Toxicology, College of Pharmacy, Qassim University, Qassim 51452, Saudi Arabia

**Keywords:** graphene oxide flakes, GOFs, gold nanoparticles, GNPs, phagocytosis, SKOV-3, granzyme, perforin, cancer cells

## Abstract

The study aimed to investigate the roles of gold nanoparticles (GNPs) and graphene oxide flakes (GOFs) as phagocytosis enhancers against cancer cells. The nanomaterials were characterized through SEM and UV-VIS absorptions. The GNPs and GOFs increased the macrophages’ phagocytosis ability in engulfing, thereby annihilating the cancer cells in both in vitro and in vivo conditions. The GNPs and GOFs augmented serine protease class apoptotic protein, granzyme, passing through the aquaporin class protein, perforin, with mediated delivery through the cell membrane site for the programmed, calibrated, and conditioned cancer cells killing. Additionally, protease inhibitor 3,4-dichloroisocoumarin (DCI) significantly reduced granzyme and perforin activities of macrophages. The results demonstrated that the GOFs and GNPs increased the activation of phagocytic cells as a promising strategy for controlling cancer cells by augmenting the cell mortality through the granzyme-perforin-dependent mechanism.

## 1. Introduction

Macrophages are cells of the immune system that are characterized by plasticity and heterogeneity [1]. In the resting state, macrophages experience different functional variations to adapt to alterations in the residing microenvironment [2]. Resting macrophages have, before stimulation, a primary phenotype that is known as M0 macrophage. When activated, these cells mainly exert the phenotypes of M1 and M2 [3]. Environmental signals can drive or polarize M0 macrophages into M1 or M2 states [4]. During emergent responses, macrophages show one of the most potent reactions characterized by their effective roles in host defense modulation, inflammation, and homeostasis [5]. These cells can control the initiation and resolution phases for innate and adaptive immune responses due to their potent capabilities to engulf bacterial cells, present antigens, and secrete cytokines [6]. The function of these cells is critically dependent on their polarization and reprogramming states. M0 macrophages can polarize into an M1 macrophage (pro-inflammatory) or M2 macrophage (anti-inflammatory) in response to various micro-milieu conditions. However, a reversal of the phenotype can also lead to the already polarized cell’s reprogramming [4].

Immune regulatory, inflammatory, and proliferative functions, in addition to activities in cellular metabolism and remodeling of tissues, are among the most prominent roles of macrophages from different polarization states. For instance, the M1 macrophages are crucial effector cells during the resistance responses against intracellular pathogens and tumor growth [7,8]. In contrast, the M2 macrophages are more involved in reactions such as immunosuppression and induction of tissue remodeling and tumor progression [9]. Moreover, the immune system’s homeostasis is influenced by the polarization and reprogramming of macrophages [5], which can confer solutions in treating the associated diseases [2]. Increased animal-based evidence demonstrated that they could cause immune system perturbation [10] and influence the course of diseases, including those of inflammation [11], allergy, and tumor [12]. As nanoparticles (NPs) are foreign to the body, macrophages have the in vivo critical roles to recognize, process, and clear them [13]. M1 or M2 macrophages show different capacities of the uptake of these particles [14]. In parallel, NPs can join the micro-milieu stimuli and contribute to prime the macrophages to polarize into one stage or another [15,16,17]. Various NPs types can affect the polarization and reprogramming of macrophages differentially [18,19], and their interaction brought much attention in both fields of toxicology research and medical application [20]. Modulating the in vivo biological influences of NPs and designing therapies that are NPs-based require a deeper understanding of the roles that these particles play in the polarization of macrophages [21,22]. The macrophages, through phagocytic engulfment, are also supported by anti-cancer preparations, i.e., trastuzumab [23] which is known to increase infiltration of macrophages into the tumor tissue with enhanced anti-cancer efficacy, have been suggested to serve as a therapeutic strategy toward enhanced killings of cancer cells. The gold nanoparticles (GNPs) were also reported [24] for cellular uptake, significantly affecting the cell proliferation activity in ovarian cancer cells, including SKOV3, and others, OVCAR5, OVCAR8. The pristine carbon quantum dots and their metal composite are also known to mediate through ovarian cancer SKOV3 cells by targeting cytokines, metalloproteinases, and cytoskeleton [25]. The role of the granzyme and its T-cell mediated releases in tumor-bearing responsive and muted-response mice models have been studied [26] regarding its immunomodulation. The CD11b, a macrophages biomarker that functionally regulates the leukocyte adhesion and migration in the inflammatory response, has been studied to show that the CD11b integrin family protein is also involved in cellular adhesion. The CD11b antibodies have been suggested with possible roles in cell-mediated cytotoxicity, chemotaxis, and phagocytosis [27]. The GNPs have reached the greatest attention in biomedical applications, with extensive research in oncology. Consequently, GNPs have become an exciting research area for cancer theranostics. Due to the unique physicochemical properties of GNPs, they have been investigated for various applications related to cancer, such as gene therapy, targeted drug delivery, radiotherapy tumor detection, and cellular bioimaging [28,29]. Graphene family materials (GFM) include several nanoparticles such as graphene oxide (GO), reduced/functionalized graphene oxide (rGO), and graphene quantum dots. Other GFM such as graphene nanoribbons, three-dimensional graphene foam, and graphene nanopores exert enormous potential in different biomedical applications. This potential arose from the exceptional physical, chemical, mechanical, and biological characteristics of these particles, along with the larger surface area that they have, the simplicity of surface functionalization, and the remarkable colloidal stability in aqueous media compared to pristine graphene [30,31,32]. The GO and rGO possess different physical and chemical characteristics, including differences in solubility, dispensability, lateral dimensions, sheet size, and the degrees and extents of their participation in redox reactions. The capacity of cellular uptake and biodegradation of these nanoparticles can be manipulated easily by using different reagents for carrying out the oxidation and reduction reactions. The differences in bioactivities levels of GO and rGO are reported in several types of bacteria and various cancer and non-cancerous cells [30,31,32]. Graphene nano-flakes or graphene nano-dots are the zero-dimensional (0-D) form of graphene while the 1-D and 2-D graphenes, carbon nanotubes, and graphene single-layer sheet, differ from each other. The graphene family materials (GFM) have interesting properties with great potential for various applications as electronic and magnetic devices. These potential applications arise because GOFs have edge states and corner states and may also be cut into a much larger variety of different shapes. GOFs can range in size from molecular to semi-infinite 2-D structures; thus, their electronic structures vary from discrete molecular levels to being band-like due to an increase in dimensions [33]. Various articles have demonstrated potential application of the GFM including the limitations [34,35]. GO and rGO demonstrate variability in batch reproducibility in addition to varying quantity of oxide, hydroxide, and epoxide thereby limiting to control the chemo- and regioselectivity of functionalization reactions. GOFs demonstrate high solubility in water, negative zeta potential, and relative ease of functionalization using carboxylate chemistry. Functionalized GOFs have demonstrated rapid blood pool clearance and renal excretion, hence can be considered as ideal candidate for theranostic drug development thus leading to its selection in the current study [35]. This study aimed to measure phagocytic cells’ activity against cancer cells, mediated by gold and graphene oxide flakes. This study proves that GNPs and GOFs induce a strong tumoricidal activity against cancer cells through the granzyme-perforin-dependent mechanism.

## 2. Materials and Methods

### 2.1. Preparation and Characterization of Nanoparticles

For preparation, procurement, and characterization of nanoparticle materials known methodology and spectro-analytical and morphology observation technique, UV-visible and scanning electron microscope (SEM) were used to measure and study the GNPs and GOFs [36].

### 2.2. Bone Marrow-Derived Macrophages

Male C57/BL6 mice (7–8 weeks old) were used as the source for the isolation of primary bone marrow-derived macrophages (BMDMs) as previously reported [37].

### 2.3. Macrophages In Vitro Tumoricidal Activity

#### 2.3.1. Immunofluorescent Assay

For this assay, phagocytic cells (BMDMs) were cultured on 4-well chamber slides for 24 h at 37 °C. Ovarian cancer cells (SKOV-3) (kindly provided by the Iraqi Center for Cancer and Medical Genetic Research, Mustansiriyah University, Baghdad, Iraq) labeled with eFluor 670–labeled were added to the BMDMs in the absence and presence of GNPs, and GOFs alone and combined at concentration 10 µg/mL. The samples were incubated for 1 h at 37 °C, the media was removed, and fresh media was added to cultured cells for 30 min, and cells were washed three times with sterile PBS. Cells were fixed using 4% formaldehyde for 30 min at 4 °C followed by staining with primary antibody using FITC–anti-CD11b at concentration 1 µg/mL for 60 min at 4 °C. Cells were washed three times using PBS for removing the unbound primary antibody. The cells were treated with secondary antibody Alexa 488-labeled anti-mouse IgG and Alexa 568-labeled anti-goat IgG Abs (Invitrogen Life Technologies, Gibco, Waltham, MA, USA) concentration 2 µg/mL at room temperature for 2 h. Finally, the phagocytic and cancer cells were examined using a confocal microscope. The SKOV-3 was stained in red color and localized in the green color of phagocytic cells (BMDMs). Finally, the samples were viewed using Meta 510 software on an LSM510 Meta Confocal microscope (Carl Zeiss, Oberkochen, Germany). The percentage of phagocytosis was calculated by phagocytic index using the following formula:

(1) $$Phagocytic\;index=\left[\left(\frac{a\;}{b}*\frac{c\;}{b}\right)*100\right]$$

where, *a* refers to the total number of engulfed cells, *b* refers to the total number of counted macrophages, and *c* refers to the number of macrophages containing engulfed cells. For this experiment, 100 cells were counted, and each experiment was performed in triplicates.

#### 2.3.2. Flow Cytometry Assay

SKOV-3 ovarian cancer cells labeled with cell proliferation dye eFluor 670 were used to identify SKOV-3 cells. The cells were then incubated with phagocytic cells at a 1:2 ratio in the presence and absence of 10 µg/mL of GNPs, and GOFs for 60 min at 37 °C. The cells were then stained with FITC–anti-CD11b for 2 h at 4 °C. A flow cytometry assay program was used to analyze the results [38].

### 2.4. Tumoricidal Activity of Macrophage Cells In Vivo

The tumoricidal activity of macrophages in the peritoneal cavity was performed according to [39]. Briefly, GNPs and GOFs were given at a dose of 500 µg/kg for three days. Ehrlich cancer cells were injected intraperitoneally into mice at (2 × 10^6^/mice). The negative group was injected with 250 µL of PBS, and the positive control was injected with Ehrlich cancer cells at a concentration of 2 × 10^6^/mice. Mice were sacrificed on day 14. The external abdominal region was inoculated with 3 mL of sterile saline. After collecting, the peritoneal cells were counted and fixed using 4% paraformaldehyde, followed by Romanowski staining after cytocentrifugation [40].

### 2.5. Isolation of Splenic Macrophage

Ten-week-old mice were immunized with the adjuvant containing killed tuberculosis germs. After 7 days, phagocytic cells were isolated from the spleen using Histopaque-1083 and incubated in plastic dishes for 1 h at 37 °C. Then, adherent cells were collected for the next experiment [41]. For granzyme activity, the phagocytic cells were pre-treated with GNPs, and GOFs at a concentration 10 µg/mL in the presence and absence of 100 µM DCI. Cells were washed two times with PBS then stained with rabbit anti-mouse granzyme B APC conjugated monoclonal antibody. Cells were fixed with a flow cytometry fixation buffer then permeabilized by permeabilization buffer. A flow cytometry assay program was used to analyze the results.

### 2.6. Statistical Analysis

Graph-Pad Prism was applied to analyze the data (three replicates). The results are represented as mean ± S.E.M. Differences were regarded as significant at *p* ≤ 0.05 [42].

## 3. Results and Discussion

### 3.1. Characterization of GNPs and GOFs

**Figure 1:**
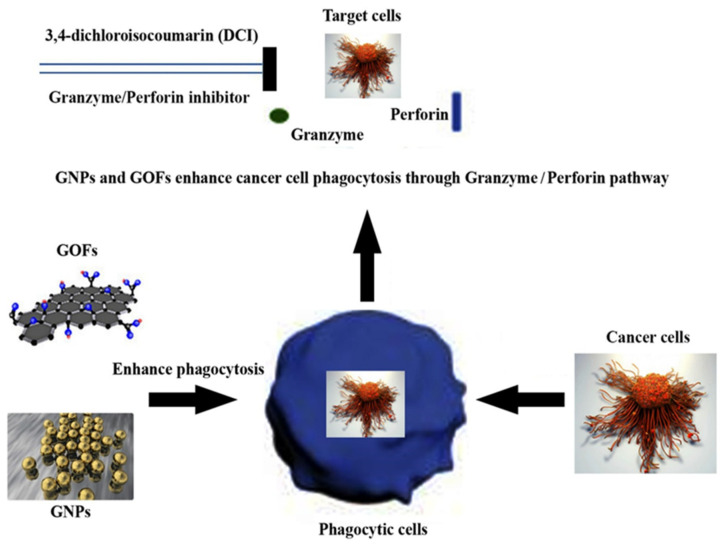
Schematic diagram of GNPs and GOFs enhancing cancer cell phagocytosis through granzyme/perforin-mediated pathway.

**Figure 2:**
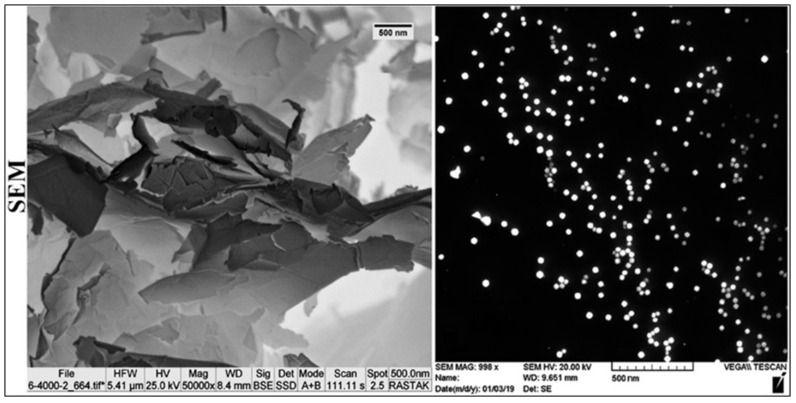
Scanning electron microscope (SEM) images of GOFs and GNPs. GNPs shape is spherical with a size of approximately 20–30 nm while GOFs size ranged approximately 9–13 nm.

The current study demonstrates phagocytic cells’ activity against cancer cells, mediated by GNPs and GOFs (Figure 1). The GNPs and GOFs were analyzed by using UV-visible (UV-VIS) spectroscopic analysis between 200 and 1100 nm wavelengths for their absorption values and were found to exhibit the λmax absorption values at almost 525 nm for GNPs, and 310 nm for GOFs. The morphology of GNPs and GOFs was studied using SEM (Figure 2) and was found to be spherical for the GNPs with a size range of 20–30 nm and almost 9–13 nm and flakes for the GOFs.

### 3.2. Evaluation of GNPs and GOFs Role in BMDMs Phagocytic Activity on SKOV-3 Cells

**Figure 3:**
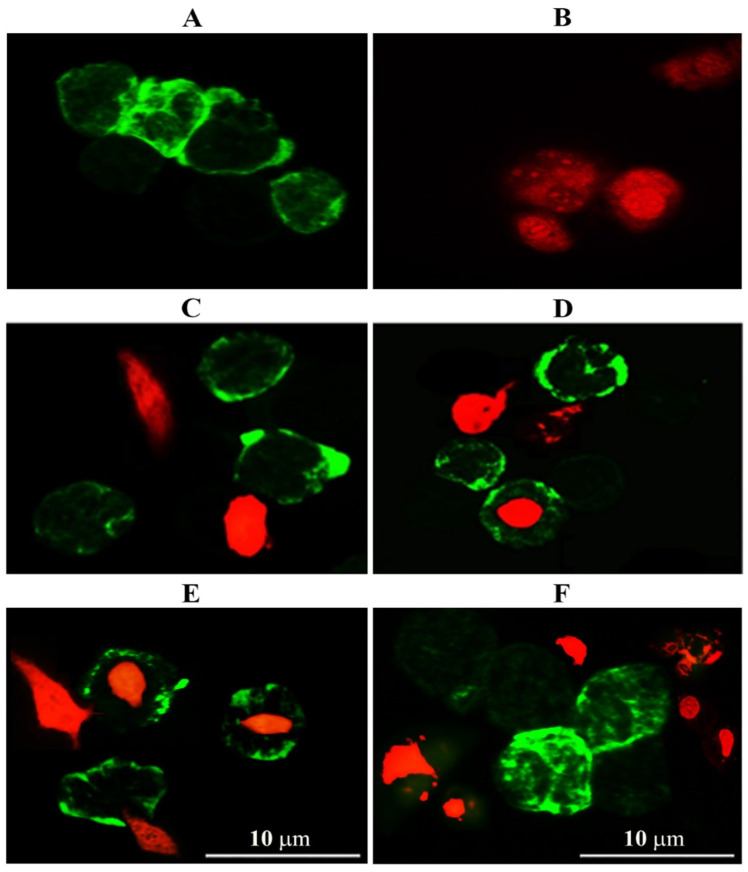
GNPs and GOFs increase the tumoricidal activity of bone marrow-derived macrophages (BMDMs) cells. (**A**) Control untreated BMDMs. (**B**) Ovarian cancer cells (SKOV-3) cells. (**C**) BMDMs engulf cancer cells. (**D**) BMDMs engulfment cancer cells in the presence of GNPs at concentration 10 µg/mL. (**E**) BMDMs engulfment cancer cells in the presence of GOFs at concentration 10 µg/mL. (**F**) BMDMs engulfment cancer cells in the presence of both GNPs and GOFs at concentration 10 µg/mL. Scale bar 10 µm.

Phagocytic cells are a part of the immune system characterized by plasticity and heterogeneity. We tested whether GNPs and GOFs could enhance ovarian cancer SKOV-3 cells killing using BMDMs as effector cells and the SKOV-3 cells as target cells. Fluorescent dye eFluor 670–labeled SKOV-3 cells were used as target cells and incubated with BMDMs cells as effector cells at a ratio of 2:1 effector: target (E: T) cells in the presence of GNPs and GOFs at concentration 10 µg/mL. The BMDMs were stained with CD11b-FITC. Cells with dual colors were detected by confocal fluorescence imaging. First, BMDMs and SKOV-3 cells images were captured separately. Secondly, in the presence of GNPs and GNPs at a concentration of 10 µg/mL, red SKOV-3 cells were observed clearly inside the green-labeled BMDMs cells (Figure 3).

**Figure 4:**
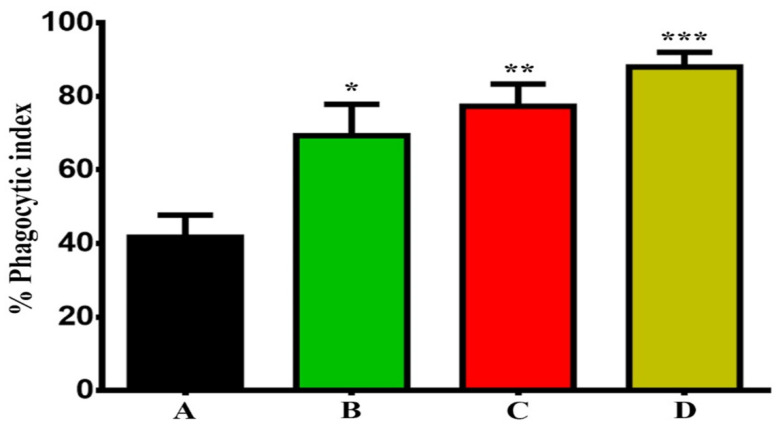
GNPs and GOFs increased the phagocytic index of cancer cells by BMDMs. (**A**) BMDMs engulfment of cancer cells. (**B**) BMDMs engulfment of cancer cells in the presence of GNPs at concentration 10 µg/mL. (**C**) BMDMs engulfment of cancer cells in the presence of GOFs at concentration 10 µg/mL. (**D**) BMDMs engulfment of cancer cells in the presence of both GNPs and GOFs at concentration 10 µg/mL. The data are represented as mean ± S.E.M. * *p* < 0.05, ** *p* < 0.01, *** *p* < 0.001 compared to the untreated BMDMs cells.

An increase in phagocytosis of cancer cells (SKOV-3) by BMDMs was demonstrated in the presence of GNPs and GOFs as an enhancer for phagocytic cells. Then, the phagocytic index of BMDMs was measured in the presence and absence of GNPs, and GOFs (Figure 4). The BMDMs showed more potent phagocytosis of SKOV-3 cells in the presence of nanoparticle materials.

**Figure 5:**
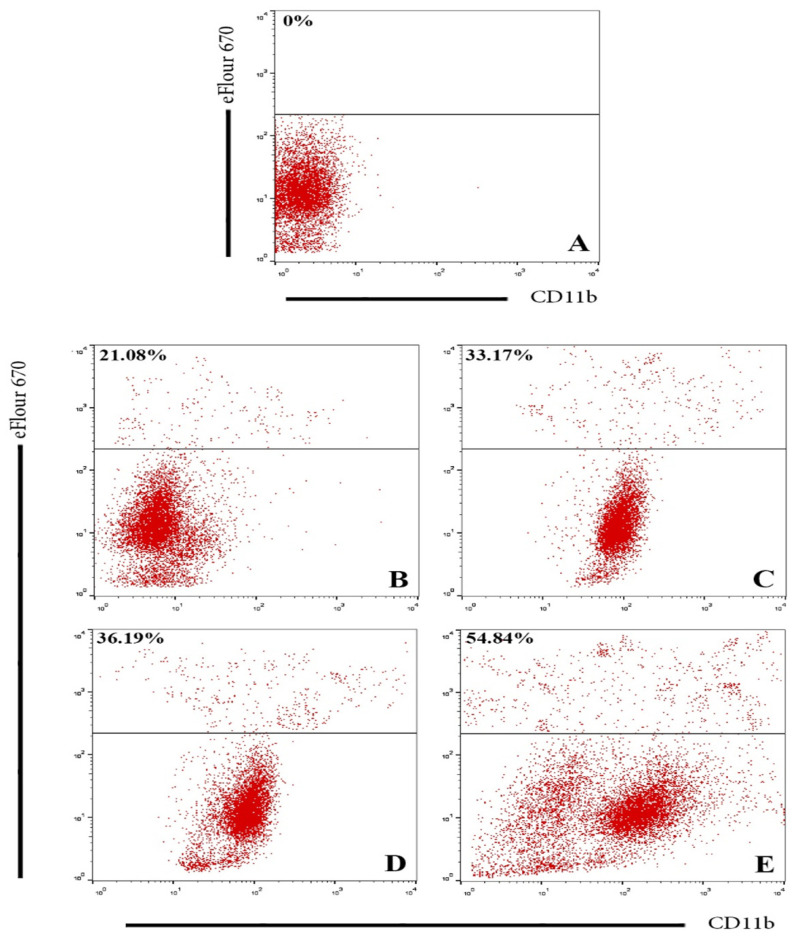
Flow cytometry analysis of GNPs- and GOFs-mediated phagocytosis of SKOV-3 cells by BMDMs. BMDMs cells were gated on CD11b+ and the double-positive population (indicating phagocytized cancer cells by BMDMs). (**A**) Control untreated BMDMs. (**B**) BMDMs engulfment of cancer cells. (**C**) BMDMs engulfment of cancer cells in the presence of GNPs. (**D**) BMDMs engulfment of cancer cells in the presence of GOFs. (**E**) BMDMs engulfment of cancer cells in the presence of both GNPs and GOFs at a concentration of 10 µg/mL.

To investigate and study the population of BMDMs with SKOV-3 phagocytosis, we gated CD11b+ cells and then measured the percentage of BMDMs with double staining (Figure 5). The results showed a significant increase in the CD11b+/eFlour 670+ cell population in BMDMs pre-treated with GNPs and GOFs compared with the control group, BMDMs alone without nanoparticles, taken together, the results demonstrated that the GNPs and GOFs increased ovarian cancer cells (SKOV-3) phagocytized by BMDMs.

### 3.3. GNPs and GOFs Increase the In Vivo Tumoricidal Activity

**Figure 6:**
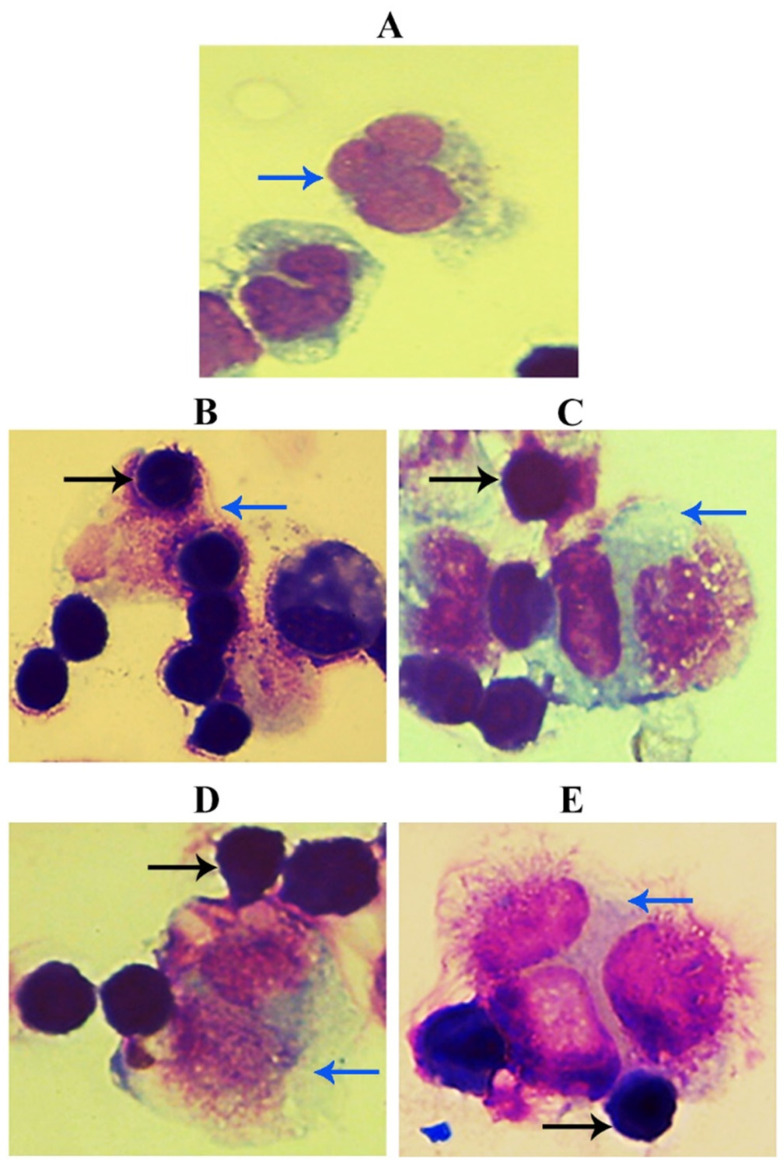
GNPs and GOFs increase the in vivo tumoricidal activity of macrophages. (**A**) Control untreated peritoneal macrophages. (**B**) Peritoneal macrophages injected with Ehrlich cancer cells. (**C**) Peritoneal macrophages injected with Ehrlich cancer cells in the presence of GNPs at concentration 10 µg/mL. (**D**) Peritoneal macrophages injected with Ehrlich cancer cells in the presence of GOFs at concentration 10 µg/mL. (**E**) Peritoneal macrophages injected with Ehrlich cancer cells in the presence of GNPs and GOFs at concentration 10 µg/mL. The blue arrow indicates macrophage cells, while the black arrow indicates Ehrlich ascites tumor cells. Magnification power 100×.

**Figure 7:**
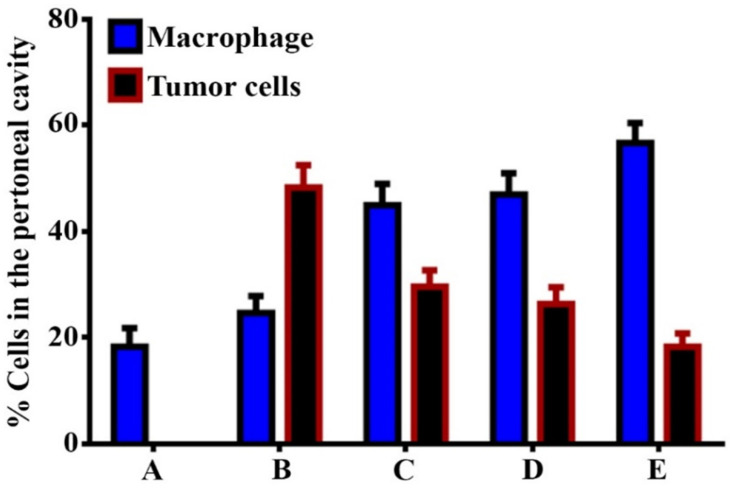
Percentage of the macrophages and tumor cells present in the peritoneal cavity. (**A**) macrophages cells in the control group. (**B**) macrophages cells and Ehrlich tumor cells. (**C**) macrophages and Ehrlich tumor cells in the presence of GNPs at concentration 10 µg/mL. (**D**) macrophages and Ehrlich tumor cells in the presence of GOFs at concentration 10 µg/mL. (**E**) macrophages and Ehrlich tumor cells in the presence of GNPs and GOFs at concentration 10 µg/mL. The data are represented as mean ± SEM.

The tumoricidal activity was confirmed using an animal model. Figure 6 and Figure 7 demonstrate the distribution of peritoneal macrophages pre-treated with GNPs and GOFs containing many cytoplasmic vacuoles. Pre-treatment with nanoparticle materials induced the biological, physiological, and functional activities of BMDMs against Ehrlich ascites tumor cells. This study showed that GNPs and GOFs as phagocytosis inducer materials could be the most potent effector and significantly increased the anti-cancer mechanism in vivo model. Nanomaterials’ ability to induce phagocytic cell activation could be related to their ability to induce and increase reactive oxygen species release (ROS). ROS can enter mitochondria, leading to macrophages’ activation and induction and stimulation of some biological and inflammatory pathways.

Nevertheless, the mechanism of tumor cell death that is associated with cytokine production is not clearly understood. Taken together, the results showed that the treatment with GOFs and GNPs alone or as combined lead to an increase in the tumoricidal activity of macrophages in the mice against Ehrlich ascites tumor cells compared with the control group. The augment of macrophage activity may refer to nanoparticles’ ability to reduce the growth of tumor cells. Thus, GOFs and GNPs may directly or indirectly affect tumor cells by stimulating the host cells, such as macrophages, which lead to induced production of cytokines such as IL-1 (interleukin-1), IL-6, and TNF (tumor necrosis factor). Some of these cytokines directly affect tumor cells or have the ability to induce cytotoxic and natural killer cells. Besides, these cytokines may induce CRP (C-reactive protein) production and complement factor C3 that would act as opsonins factor against tumor cells [43]. An additional experiment involving the granzyme perforin pathway complemented the anti-tumor activities.

### 3.4. GNPs and GOFs Augmented Macrophages Kill Tumor Cells through a Granzyme-Perforin Pathway

**Figure 8:**
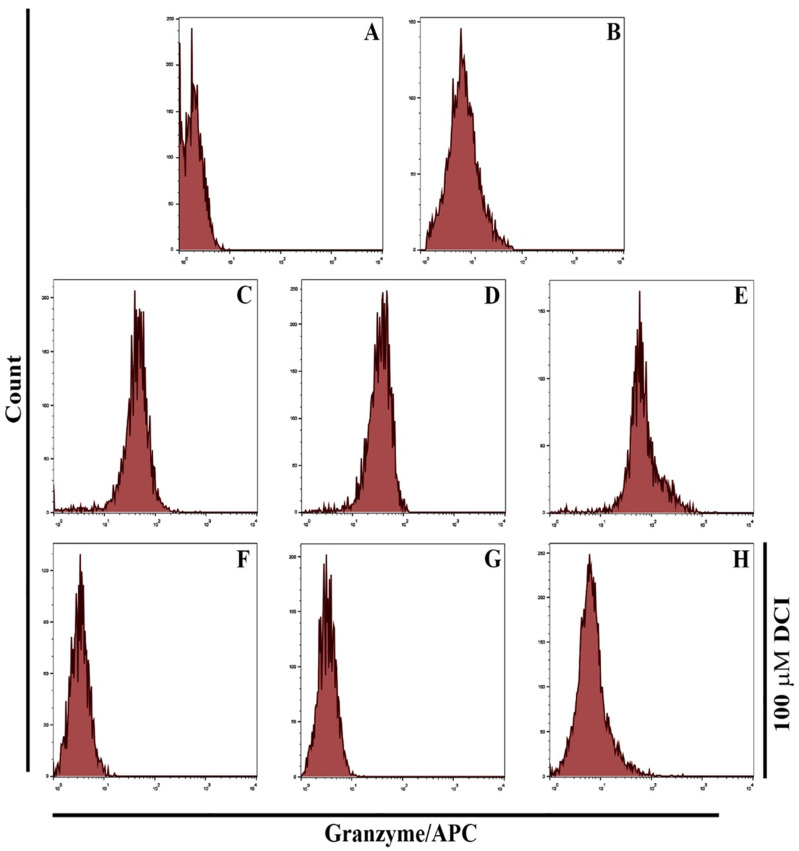
GNPs and GOFs increase the tumoricidal activity of macrophages through the granzyme–perforin pathway. (**A**) Control un-treated macrophages. (**B**) macrophages with cancer cells (**C**) macrophages and cancer cells in the presence of GNPs at a concentration 10 µg/mL. (**D**) macrophages and cancer cells in the presence of GOFs at a concentration 10 µg/mL. (**E**) macrophages cancer cells in the presence of GNPs and GOFs at a concentration 10 µg/mL. Figures (**F**–**H**) are similar to figures (**C**–**E**) in the presence of 100 µM of DCI (Ganzyme inhibitor). APC, allophycocyanin.

This study showed that the granzyme B was spread in the cytoplasm of splenic macrophages, as seen in Figure 8. Splenic phagocytic cells that are pre-treated with GNPs and GOFs exhibited more granzyme B signaling. Cytolytic enzymes are critical mediators of anti-tumor immunity. Combination of granzymes and perforin expressed by various immune cells alongside markers of αβ and γδ T-cell maturation is indicative of the immunological involvements. The expression of CD68 (Cluster of Differentiation 68, a protein) in macrophages, and the use of antagonistic, 3,4-dichloroisocoumarin (DCI), as protease inhibitors for granzyme, and degranulation process of the cytolytic granules, was confirmed for the antagonistic action of inhibiting cytotoxic activities of the chosen splenic macrophages. The granzymes trigger apoptotic pathways while perforin protects the mice against developing B-cell lymphoma and another carcinogens-induced sarcoma. The pathway can influence immunopathology, though, in an organ-specific manner, reduced antigen has been observed for malignant cells. Many alternate pathways can be involved; however, the immune cells’ effective pathway choice involves interferon-γ, perforin-granzyme, and FasL, a type-II transmembrane protein belonging to the family [44,45]. To investigate if granzyme B and perforin play an unimportant role in tumor cells’ killing mechanism, we used DCI as protease inhibitors to inhibit granzyme activities in the cytolytic granules’ degranulation process. The results show the ability of DCI to inhibit cytotoxic activities of splenic macrophages. The results showed very clear weak signaling of granzyme in GNPs and GOFs pre-treated macrophages in the presence of 100 µM of DCI.

## 4. Conclusions

In the present study, GNPs and GOFs were used as phagocytosis inducers, and the role of these nanomaterials as phagocytosis inducer agents was investigated using in vitro and in vivo models. The GNPs and GOFs were up-regulated and mediated the phagocytosis killing of the cancer cells. The present work proves the GNPs and GOFs mediated and increased macrophages tumoricidal activity through granzyme-perforin dependent mechanism, a viable option for cancer cell killing. The study has prospects to develop as immunotherapy against cancers, and the role of nanoparticles is important to investigate the immunological aspects involved in the process. Moreover, the molecular roles of perforin and granzyme, separately, need to be understood. The molecular mechanistic details the trigger for the choice of the granzyme-perforin pathway, and nanomaterials induction to the cancer cell killings need to be explored.

## Data Availability

Not applicable.

## References

[1] Taylor P.R., Martinez-Pomares L., Stacey M., Lin H.-H., Brown G.D., Gordon S. (2005). Macrophage receptors and immune recognition. Annu. Rev. Immunol..

[2] Mills C.D., Kincaid K., Alt J.M., Heilman M.J., Hill A.M. (2000). M-1/M-2 Macrophages and the Th1/Th2 Paradigm. J. Immunol..

[3] Giovanni M., Yue J., Zhang L., Xie J., Ong C.N., Leong D.T. (2015). Pro-inflammatory responses of RAW264.7 macrophages when treated with ultralow concentrations of silver, titanium dioxide, and zinc oxide nanoparticles. J. Hazard. Mater..

[4] Martinez F.O. (2011). Regulators of macrophage activation. Eur. J. Immunol..

[5] Laskin D.L., Sunil V.R., Gardner C.R., Laskin J.D. (2011). Macrophages and Tissue Injury: Agents of Defense or Destruction?. Annu. Rev. Pharmacol. Toxicol..

[6] Wynn T.A., Chawla A., Pollard J.W. (2013). Macrophage biology in development, homeostasis and disease. Nature.

[7] Zanganeh S., Hutter G., Spitler R., Lenkov O., Mahmoudi M., Shaw A., Pajarinen J.S., Nejadnik H., Goodman S., Moseley M. (2016). Iron oxide nanoparticles inhibit tumour growth by inducing pro-inflammatory macrophage polarization in tumour tissues. Nat. Nanotechnol..

[8] Hill A.A., Bolus W.R., Hasty A.H. (2014). A decade of progress in adipose tissue macrophage biology. Immunol. Rev..

[9] Roszer T. (2015). Understanding the Mysterious M2 Macrophage through Activation Markers and Effector Mechanisms. Mediat. Inflamm..

[10] Getts D.R., Shea L.D., Miller S.D., King N.J.C. (2015). Harnessing nanoparticles for immune modulation. Trends Immunol..

[11] Cho W.-S., Duffin R., Thielbeer F., Bradley M., Megson I.L., MacNee W., Poland C.A., Tran C.L., Donaldson K. (2012). Zeta Potential and Solubility to Toxic Ions as Mechanisms of Lung Inflammation Caused by Metal/Metal Oxide Nanoparticles. Toxicol. Sci..

[12] Oberdörster G., Ferin J., Lehnert B.E. (1994). Correlation between particle size, in vivo particle persistence, and lung injury. Environ. Health Perspect..

[13] Brandenberger C., Rowley N.L., Jackson-Humbles D.N., Zhang Q., Bramble L.A., Lewandowski R.P., Wagner J.G., Chen W., Kaplan B.L., Kaminski N.E. (2013). Engineered silica nanoparticles act as adjuvants to enhance allergic airway disease in mice. Part. Fibre Toxicol..

[14] Dobrovolskaia M.A., McNeil S.E. (2007). Immunological properties of engineered nanomaterials. Nat. Nanotechnol..

[15] MacParland S.A., Tsoi K.M., Ouyang B., Ma X.-Z., Manuel J., Fawaz A., Ostrowski M.A., Alman B.A., Zilman A., Chan W.C.W. (2017). Phenotype Determines Nanoparticle Uptake by Human Macrophages from Liver and Blood. ACS Nano.

[16] Lucarelli M., Gatti A.M., Savarino G., Quattroni P., Martinelli L., Monari E., Boraschi D. (2004). Innate defence functions of macrophages can be biased by nano-sized ceramic and metallic particles. Eur. Cytokine Netw..

[17] Laskar A., Eilertsen J., Li W., Yuan X.-M. (2013). SPION primes THP1 derived M2 macrophages towards M1-like macrophages. Biochem. Biophys. Res. Commun..

[18] Bartneck M., Ritz T., Keul H.A., Wambach M., Bornemann J., Gbureck U., Ehling J., Lammers T., Heymann F., Gassler N. (2012). Peptide-Functionalized Gold Nanorods Increase Liver Injury in Hepatitis. ACS Nano.

[19] Fuchs A.-K., Syrovets T., Haas K.A., Loos C., Musyanovych A., Mailänder V., Landfester K., Simmet T. (2016). Carboxyl- and amino-functionalized polystyrene nanoparticles differentially affect the polarization profile of M1 and M2 macrophage subsets. Biomaterials.

[20] Talekar M., Tran T.-H., Amiji M. (2015). Translational Nano-Medicines: Targeted Therapeutic Delivery for Cancer and Inflammatory Diseases. AAPS J..

[21] Oh N., Park J.H. (2014). Endocytosis and exocytosis of nanoparticles in mammalian cells. Int. J. Nanomed..

[22] Cai W. (2008). Applications of gold nanoparticles in cancer nanotechnology. Nanotechnol. Sci. Appl..

[23] Shi Y., Fan X., Deng H., Brezski R.J., Rycyzyn M., Jordan R.E., Strohl W.R., Zou Q., Zhang N., An Z. (2015). Trastuzumab Triggers Phagocytic Killing of High HER2 Cancer Cells In Vitro and In Vivo by Interaction with Fcγ Receptors on Macrophages. J. Immunol..

[24] Kumar D., Mutreja I., Chitcholtan K., Sykes P. (2017). Cytotoxicity and cellular uptake of different sized gold nanoparticles in ovarian cancer cells. Nanotechnology.

[25] Chen D., Li B., Lei T., Na D., Nie M., Yang Y., Congjia, Xie, He Z., Wang J. (2021). Selective mediation of ovarian cancer SKOV3 cells death by pristine carbon quantum dots/Cu_2_O composite through targeting matrix metalloproteinases, angiogenic cytokines and cytoskeleton. J. Nanobiotechnology.

[26] Nguyen A., Ramesh A., Kumar S., Nandi D., Brouillard A., Wells A., Pobezinsky L., Osborne B., Kulkarni A.A. (2020). Granzyme B nanoreporter for early monitoring of tumor response to immunotherapy. Sci. Adv..

[27] Christensen J.E., Andreasen S.Ø., Christensen J.P., Thomsen A.R. (2001). CD11b expression as a marker to distinguish between recently activated effector CD8+ T cells and memory cells. Int. Immunol..

[28] Ghosh P., Han G., De M., Kim C.K., Rotello V.M. (2008). Gold nanoparticles in delivery applications. Adv. Drug Deliv. Rev..

[29] Hainfeld J.F., Dilmanian F.A., Zhong Z., Slatkin D.N., Kalef-Ezra J.A., Smilowitz H.M. (2010). Gold nanoparticles enhance the radiation therapy of a murine squamous cell carcinoma. Phys. Med. Biol..

[30] Al Rugaie O., Jabir M., Kadhim R., Karsh E., Sulaiman G.M., Mohammed S.A.A., Khan R.A., Mohammed H.A. (2021). Gold Nanoparticles and Graphene Oxide Flakes Synergistic Partaking in Cytosolic Bactericidal Augmentation: Role of ROS and NOX2 Activity. Microorganisms.

[31] Bhattacharya M. (2016). Polymer Nanocomposites A Comparison between Carbon Nanotubes, Graphene, and Clay as Nanofillers. Materials.

[32] Gurunathan S., Han J.W., Eppakayala V., Dayem A.A., Kwon D.-N., Kim J.-H. (2013). Biocompatibility effects of biologically synthesized graphene in primary mouse embryonic fibroblast cells. Nanoscale Res. Lett..

[33] Barnard A., Snook I. (2008). Thermal stability of graphene edge structure and graphene nanoflakes. J. Chem. Phys..

[34] Lamb J., Fischer E., Rosillo-Lopez M., Salzmann C.G., Holland J.P. (2019). Multi-functionalised graphene nanoflakes as tumour-targeting theranostic drug-delivery vehicles. Chem. Sci..

[35] Jampilek J., Kralova K. (2021). Advances in Drug Delivery Nanosystems Using Graphene-Based Materials and Carbon Nanotubes. Materials.

[36] Kadhim R.J., Karsh E.H., Jabir M.S. (2020). Anti-inflammatory activity of gold and graphene oxide nanoparticles in-vitro study. Proceedings of the 2nd International Conference on Materials Engineering & Science (IConMEAS 2019).

[37] Taqi Z.J., Abdul-Wahed H.E., AL-Saadi H.K., Jabir M.S. (2020). Potential activity of silver nanoparticles synthesized by Iraqi propolis on phagocytosis. Proceedings of the 2nd International Conference on Materials Engineering & Science (IConMEAS 2019).

[38] Al-Salman H.N.K., Ali E.T., Jabir M., Sulaiman G.M., Al-Jadaan S.A.S. (2020). 2-Benzhydrylsulfinyl-N-hydroxyacetamide-Na extracted from fig as a novel cytotoxic and apoptosis inducer in SKOV-_3_ and AMJ-_13_ cell lines via P_53_ and caspase-8 pathway. Eur. Food Res. Technol..

[39] Sulaiman G.M., Waheeb H.M., Jabir M.S., Khazaal S.H., Dewir Y.H., Naidoo Y. (2020). Hesperidin Loaded on Gold Nanoparticles as a Drug Delivery System for a Successful Biocompatible, Anti-Cancer, Anti-Inflammatory and Phagocytosis Inducer Model. Sci. Rep..

[40] Jabir M.S., Ritchie N.D., Li D., Bayes H.K., Tourlomousis P., Puleston D., Evans T.J. (2014). Caspase-1 cleavage of the TLR adaptor TRIF inhibits autophagy and β-interferon production during Pseudomonas aeruginosa infection. Cell Host Microbe..

[41] Baba T., Iwasaki S., Maruoka T., Suzuki A., Tomaru U., Ikeda H., Ishizu A. (2008). Rat CD4^+^ CD8^+^ macrophages kill tumor cells through an NKG2D-and granzyme/perforin-dependent mechanism. J. Immunol..

[42] Khashan K.S., Abdulameer F.A., Jabir M.S., Hadi A.A., Sulaiman G.M. (2020). Anticancer activity and toxicity of carbon nanoparticles produced by pulsed laser ablation of graphite in water. Adv. Nat. Sci. Nanosci. Nanotechnol..

[43] Khan F., Niaz K., Maqbool F., Ismail Hassan F., Abdollahi M., Nagulapalli Venkata K.C., Bishayee A. (2016). Molecular targets underlying the anti-cancer effects of quercetin: An update. Nutrients.

[44] Chattopadhyay P.K., Betts M.R., Price D.A., Gostick E., Horton H., Roederer M., Rosa S.C. (2008). De The cytolytic enzymes granyzme A, granzyme B, and perforin: Expression patterns, cell distribution, and their relationship to cell maturity and bright CD57 expression. J. Leukoc. Biol..

[45] Trapani J.A., Smyth M.J. (2002). Functional significance of the perforin/granzyme cell death pathway. Nat. Rev. Immunol..

